# Rethinking Urbanicity: Conceptualizing Neighborhood Effects on Women’s Mental Health in Kampala’s Urban Slums

**DOI:** 10.3390/ijerph23010041

**Published:** 2025-12-28

**Authors:** Monica H. Swahn, Peter Kalulu, Hakimu Sseviiri, Josephine Namuyiga, Jane Palmier, Revocatus Twinomuhangi

**Affiliations:** 1 Department of Epidemiology, School of Public Health, Virginia Commonwealth University, Richmond, VA 23219, USA; 2 Division of Infectious Diseases, John T. Milliken Department of Medicine, School of Medicine, Washington University in St. Louis, St. Louis, MO 63110, USA; kalulu@wustl.edu; 3 Department of Geography, Geoinformatics and Climatic Sciences, College of Agricultural and Environmental Sciences, Makerere University, Kampala 7062, Ugandartwinomuhangi@gmail.com (R.T.); 4 Uganda Youth Development Link, Kampala 12659, Uganda; 5 School of Public Health, Virginia Commonwealth University, Richmond, VI 23219, USA

**Keywords:** urbanicity, mental health, urban slums, women’s health, Kampala, Uganda

## Abstract

Urbanicity is a recognized determinant of mental health, yet conventional measures such as population density or the rural–urban divide often fail to capture the complex realities of informal settlements in low- and middle-income countries. This paper conceptualizes neighborhood effects through the lived experiences of young women in Kampala, Uganda, drawing on participatory research from the NIH-funded TOPOWA study. Using community mapping and Photovoice, participants identified neighborhood features that shape wellbeing, including sanitation facilities, drainage systems, alcohol outlets, health centers, schools, boda boda stages (motorcycle taxis), lodges, religious institutions, water sources, markets, and recreational spaces. These methods revealed both stressors—poor waste management, flooding, violence, gendered harassment, crime, and alcohol-related harms—and protective resources, including education, places of worship, health centers, social networks, identity, and sports activities. We argue that urbanicity in slum contexts should be understood as a multidimensional construct encompassing deprivation, fragmentation, exclusion, and resilience. This reconceptualization advances conceptual clarity, strengthens the validity of mental health research in low-resource settings, and informs interventions that simultaneously address structural risks and promote community assets. The case of Kampala demonstrates how participatory evidence can reshape the understanding of neighborhood effects with implications, for global mental health research and practice.

## 1. Introduction

Urbanicity has long been recognized as a determinant of mental health [1,2,3,4,5], but the ways in which it is defined and operationalized remain uneven and contested [6,7]. In much of the global literature, urbanicity has been narrowly measured through indicators such as population density [5,7], levels of urban infrastructure [8], or the binary distinction between urban and rural residence [9,10]. These indicators, while useful for large-scale comparisons, often obscure the realities of populations living in informal and slum settlements, particularly in low- and middle-income countries (LMICs), where multiple aspects of deprivation, institutional neglect, insecurity, and social resilience coexist. A conceptual distinction in this study is that between urbanicity and deprivation. The urban health literature has associated urbanicity with characteristics such as living in an urban environment, higher population density, better infrastructure, and greater access to services [11]. In contrast, deprivation has been associated with resource deficits, including poverty, poor housing, unemployment, and inadequate access to services [12,13,14]. In high-income countries, urbanicity and deprivation may overlap, but in LMICs, they diverge. In LMICs, highly populated urban areas may not necessarily indicate greater access to services; living in urban areas may not imply ownership of material resources or the presence of public or private institutions. Understanding these differences is very important because people living in densely populated urban slums may be experiencing deprivation, which is important in understanding the risk to mental health. This is particularly problematic for women, whose health outcomes are shaped not only by material deprivation but also by gendered experiences of violence, stigma, and structural exclusion. In such environments, the urban condition cannot be reduced to the number of people per square kilometer or the presence of formal infrastructure. Instead, urbanicity represents a complex interplay of social, economic, political, technological, technical, and environmental dynamics that generate both risks and opportunities for health [15,16,17,18]. As a result, it is essential to reconsider urbanicity as a construct that is both relational and influenced by gender, emphasizing the impact of neighborhood dynamics. This includes examining how social, economic, and environmental factors interact with personal vulnerabilities to influence health outcomes [19].

Scholarship in feminist geography and gendered urban studies has long emphasized that women experience cities differently from men due to intersecting structural forces, including unequal access to public space, heightened exposure to harassment and violence, and disproportionate responsibility for caregiving and household tasks. These perspectives highlight that urban environments are not neutral but are shaped by gendered power relations, mobility constraints, and the unequal distribution of safety, resources, and opportunity. Intersectional approaches further underscore how gender combines with poverty, informality, and marginalization to produce distinct patterns of vulnerability and resilience. These insights align with and motivate our focus on young women’s lived experiences, which provide a critical lens for understanding how urbanicity is produced and felt within informal settlements in Kampala.

In Sub-Saharan Africa, rapid urban growth has dramatically reshaped the landscape of cities over the past three decades [20]. Much of this growth has occurred in unplanned informal settlements where governance, infrastructure, and public services lag far behind demographic change [21]. This has in a way redefined the dynamics of health, with informal and slum settlements emerging as critical spaces of vulnerability. Kampala, Uganda’s capital and largest city, exemplifies this trend. A significant proportion of Kampala’s residents (over 60%) live in densely packed slums where insecure housing and tenure, inadequate sanitation, poor waste disposal, high unemployment, violence, overcrowding and fragmented governance structures are daily realities [22]. Women in particular experience these urban environments in gendered ways, facing heightened vulnerability to harassment [23], intimate partner violence [24,25], and exclusion from economic opportunities [26], while also navigating disproportionate caregiving burdens within households [27]. We have captured the social drivers of health risks in previous work, where young women have outlined the key intersectional social injustices; issues with inadequate housing and sanitation; the limited capacity of health care; and need for health promotion efforts that build on existing resources and strengths in the community [28]. It is very important to note that the young women also perceive their neighborhoods as sites of resilience, social creativity, spaces of hope, and collective mobilization, as residents develop networks and strategies for survival amidst adversity [29]. In fact, we find that among young women in Kampala, resilience is key in the linkages between neighborhood context (satisfaction and cohesion) and mental health outcomes [30].

The mental health implications of urbanicity are increasingly acknowledged in global health research [31]. Studies from high-income countries have highlighted the effects of neighborhood characteristics such as noise pollution [32], traffic congestion [33], air quality [34], overcrowding [3], a lack of green space [35], and crime rates on common mental disorders, including depression and anxiety [36]. Neighborhood-effects research [37], including Faris and Dunham’s seminal work in Chicago [38] and the research of others, provides strategies for how to document the spatial clustering of mental illness and how to contextualize theories such as social disorganization, which focuses on structural disadvantage, including weakened institutions, and collective efficacy, which emphasizes the importance of social cohesion [39,40,41]. Additionally, urban resilience frameworks often focus on adaptive capacity and infrastructural robustness [42]. Later scholarship emphasized the importance of social cohesion, concentrated disadvantage, and place-based inequities as determinants of mental health [43,44,45,46]. However, these existing theories and frameworks do not fully account for the presence of deprivation, institutional exclusion, spatial fragmentation, and slums in urban African cities. Therefore, when these frameworks are transposed to LMIC settings, they often fail to capture the unique stressors that shape mental health in slums. Unlike neighborhoods in high-income settings, urban slums are not the product of gradual decline but rather the outcome of rapid and unregulated urbanization combined with chronic poverty, service/infrastructure deficits, and weak governance [47].

In Kampala’s slums, conventional indicators of urbanicity, such as population density or access to green space, reveal little about the lived experience of urban environments. The neighborhoods are instead defined by intersecting conditions such as persistent flooding due to inadequate drainage, poor sanitation and waste management, widespread alcohol sales and marketing, anti-social behaviors (drug addiction and sex work/prostitution), chronic unemployment, and systemic neglect by public institutions [29,48]. These conditions may also overlap with gender-based violence, transactional sex, and climate-related shocks to create layered vulnerabilities for young women [25,49]. Mental health risks are therefore shaped not simply by density or infrastructure but by socio-structural disadvantage and the ways in which women navigate spaces of risk and resilience. There is a clear need to integrate the local realities when addressing mental health across Africa [50]. Yet, despite the growing recognition of urban health challenges, women’s mental health in the context of Kampala’s slums remains understudied and under-prioritized.

Foundational work in urban health has long emphasized that urbanicity cannot be understood solely through binary rural–urban classifications or population density measures. Seminal contributions have demonstrated that urban contexts are socially stratified, structurally unequal, and shaped by neighborhood dynamics such as social disorganization, collective efficacy, and concentrated disadvantage [2,3,4,5,6,7,14]. These frameworks, however, were developed largely in high-income countries and do not draw on participatory or gendered evidence from informal settlements in sub-Saharan Africa. Moreover, widely used indices of deprivation, such as the Area Deprivation Index, Social Deprivation Index, and Social Vulnerability Index, rely on census-based metrics that presuppose formal infrastructure, stable administrative boundaries, and standardized service systems. In informal settlements, population density does not always correlate with service access, and deprivation is experienced through institutional absence, fragmented governance, and layered spatial risks. In this paper, we extend the urban health literature by grounding the conceptualization of urbanicity in young women’s lived experiences in Kampala’s slums and proposing a multidimensional framework, incorporating deprivation, fragmentation, exclusion, and resilience, derived from participatory mapping and Photovoice activities. This approach centers informality, gender, and lived realities as key to understanding how urban environments shape mental health.

As such, this paper builds on, but diverges from, the existing theories and frameworks and seeks to reconceptualize neighborhood effects through a participatory approach and the lens of young women’s lived experiences in Kampala’s slums. The analysis and conceptual development draw on formative research from the NIH-funded Onward Project on Wellbeing and Adversity (TOPOWA), including community mapping and participatory Photovoice activities with young women in Kampala’s slum settlements. These approaches enable young women to articulate, in their own terms, the neighborhood features that shape their mental health and well-being. Their perspectives reveal both the inadequacy of conventional definitions of urbanicity and the need for a more grounded, multidimensional conceptualization of neighborhood effects that recognizes deprivation, fragmentation, exclusion, and resilience as coexisting dimensions of urban life.

The aim of this paper is therefore twofold. First, we critically examine the limitations of existing definitions of urbanicity when applied to LMIC slum contexts. Second, we propose an alternative framework for assessing neighborhood effects that is, grounded in community-driven evidence and realities from Kampala, that captures how urban environments affect women’s mental health. By situating urbanicity within the everyday life of women in slum settlements, this paper seeks to advance conceptual clarity in global urban health research and to inform interventions that address both risks and protective resources in disadvantaged urban settings. Further, by centering women’s experiences in Kampala’s slums, the paper contributes to ongoing debates related to urban health equity.

## 2. Methods

### 2.1. Study Setting

Kampala, Uganda’s capital and largest city, has experienced rapid and uneven urbanization over the past three decades [51]. Much of this growth has been absorbed into informal and slum settlements characterized by poverty, overcrowding and gender-based violence, and where infrastructure, governance, and social protection remain limited [22]. The TOPOWA study and its formative activities were conducted in slum settlements of three administrative units in Kampala city: Banda (Nakawa division), Bwaise (Kawempe division), and Makindye (Makindye division). These sites reflect the diversity of urban slum environments in Kampala and illustrate the diverse layered factors that define neighborhood life.

### 2.2. Community Mapping

**Figure 1:**
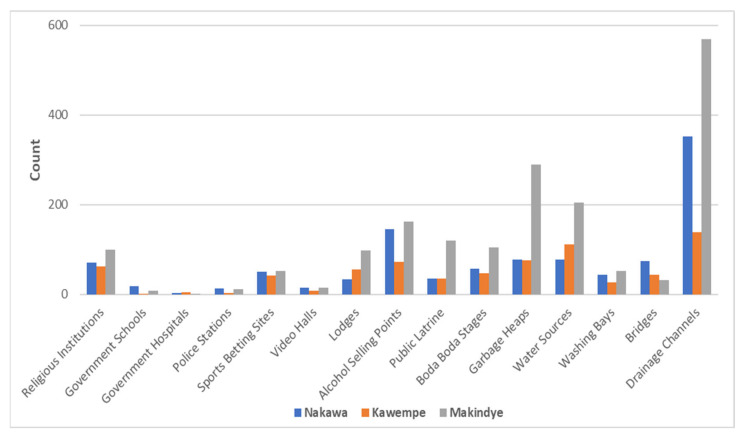
Raw counts from Community Mapping of Key Neighborhood Features across the three Study Sites in Nakawa (Banda), Kawempe (Bwaise), and Makindye.

Community mapping was undertaken in 2022 to identify neighborhood features that local residents viewed as central to wellbeing and mental health The activity focused on the communities surrounding the three Uganda Youth Development Link (UYDEL) centers and TOPOWA project study sites in Bwaise, Makindye, and Banda, drawing on 26 parishes located in these areas (12 in Makindye, 6 in Bwaise, and 8 in Banda). The selection of indicators and mapping priorities was informed through a multi-stakeholder consultative process, including engagement with the TOPOWA Youth Advisory Board and discussions with women community leaders from each parish. Women leaders were intentionally included because of their close interactions with young women around issues of health, safety, counseling, and community services, and their knowledge of neighborhood conditions. Through this participatory process, young women and community leaders identified features such as public latrines, drainage channels, alcohol selling points, schools, hospitals, boda boda stages (motorcycle taxis), lodges, police posts, religious institutions, water sources, video halls, washing bays, and gambling centers, which were then systematically geo-located by trained community enumerators. The mapping exercise provided a spatial record (Figure 1), albeit not a census, of socio-economic, and environmental features and also validated the importance of neighborhood-level determinants as defined by community members themselves. Importantly, the emphasis was not on producing an exhaustive count of features and facilities, but on highlighting the types of features that shape daily life and mental health and their relative presence within the community that served as our study site.

### 2.3. Photovoice Recruitment and Project

In parallel, a Photovoice exercise engaged young women aged 18–24 years in documenting their communities through photography (described elsewhere) [29,52,53]. Fifteen young women aged 18–24 years were recruited from three UYDEL drop-in centers located in Banda, Bwaise, and Makindye. Recruitment was conducted by UYDEL staff using outreach lists and center attendance logs, and interested women were invited to participate in the Photovoice project on mental health and well-being, which included five sessions including a final thematic review and discussion. Participants were asked to capture images of both stressors and protective resources which were then analyzed in facilitated group sessions. The Photovoice exercise culminated in the discussion of thematic categories such as poor disposal of rubbish and sanitation challenges, insecurity, alcohol and drug use, schools, worship places, health centers, markets, sports, family life, and forms of community development such as electricity and transport networks. This participatory approach enabled women to articulate, in their own voices, how urbanicity is lived and experienced. As compensation for transportation and time, participants received USD 56 total upon completing all five project sessions.

### 2.4. Data Analysis

This manuscript is conceptual in nature; therefore, the analytic approach emphasized the organization and synthesis of participant-generated material to support the development of a broader framework for understanding urbanicity in informal settlements. The research team first compiled outputs from the community mapping exercises and Photovoice narratives and organized them into descriptive categories corresponding to features that participants identified as central to their daily lives, including sanitation challenges, drainage systems, alcohol outlets, schools, worship spaces, health centers, markets, transport nodes, and social spaces (as documented in prior TOPOWA Photovoice work [29,52]).

Through iterative team discussions, these descriptive categories were reviewed and grouped into broader domains that reflected how participants collectively characterized their neighborhoods. Feedback from the community advisory board further guided the interpretation to ensure alignment with the lived experiences of young women in the study communities. Consistent with the conceptual aims of this manuscript, we did not apply formal thematic analysis procedures, calculate intercoder reliability, or conduct inferential triangulation. Instead, our analytic focus remained on synthesizing participant insights into a coherent interpretive framework aligned with participatory approaches used previously in Kampala [29,52].

Geospatial mapping was incorporated to visually illustrate the spatial distribution of selected features identified during community mapping. GPS coordinates collected by trained community enumerators were processed and mapped by the team’s geospatial specialist using ArcGIS Version 10.8 software, drawing on publicly available administrative boundary shapefiles and geospatial layers from Kampala Capital City Authority (KCCA), the Makerere University Centre for Climate Change Research and Innovations (MUCCRI), and the Urban Action Lab (UAL) (as used in prior work [17]). These maps were used descriptively to depict patterns such as the clustering of drainage channels, illegal waste disposal sites, alcohol outlets, and limited protective infrastructure. Because the mapping was participatory and not census-based, the spatial outputs were not intended for statistical inference but rather to visually support and contextualize participants’ observations.

The domains derived from the participatory material and descriptive mapping were subsequently synthesized into four higher-order dimensions—deprivation, fragmentation, exclusion, and resilience—that form the conceptualization of urbanicity proposed in this manuscript. This approach reflects the primary aim of the study: to generate a grounded, context-specific conceptual framework informed by participatory methods and lived experience rather than to conduct a traditional qualitative or geospatial analytic procedure.

## 3. Results

The categories and themes identified through both the community mapping and photovoice are presented in Figure 1 and Table 1. These domains reflect the ways in which young women and community members themselves defined the environmental and social features of their neighborhoods, emphasizing sanitation and infrastructure, health and services, economic and workspaces, social and cultural life, security and risk, and community dynamics.

### 3.1. Risks: Deprivation and Fragmentation

**Figure 2:**
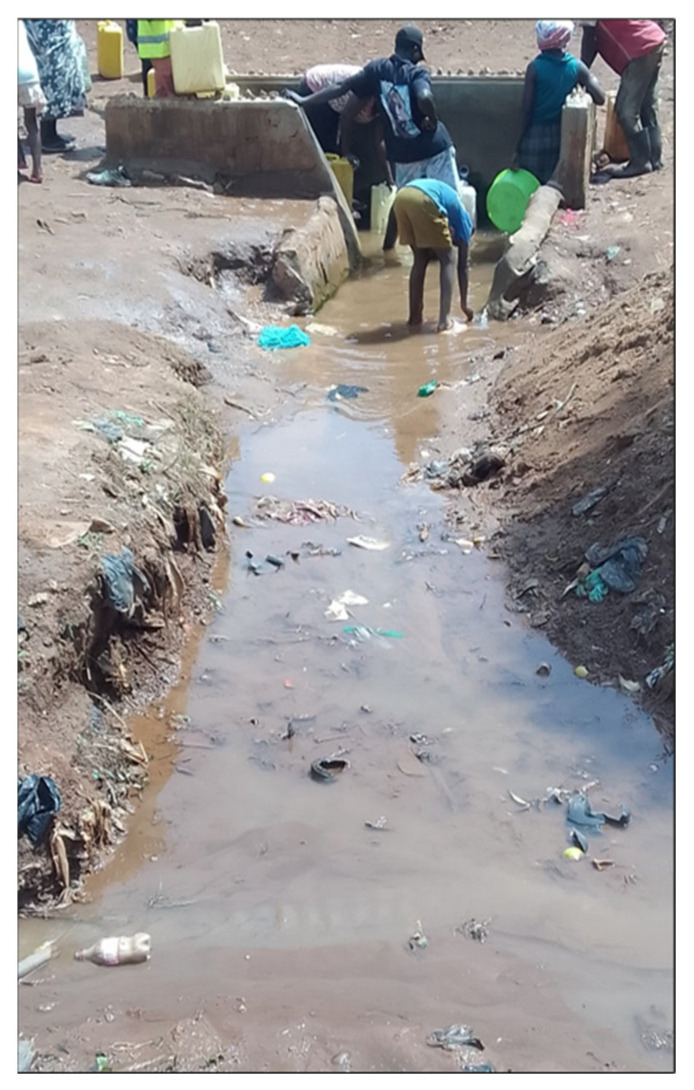
Picture of a natural spring taken during community mapping in Bwaise.

**Figure 3:**
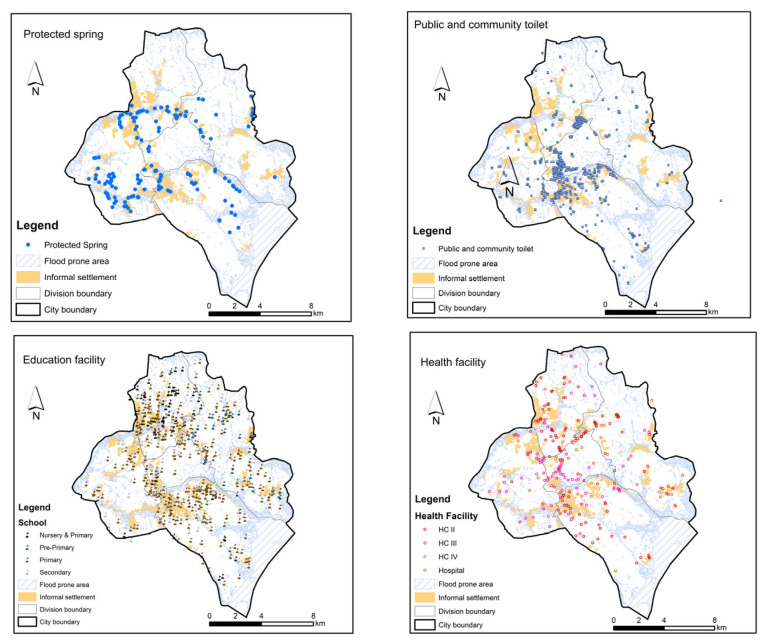
Geospatial manifestation of fragmentation, flood vulnerability and deprivation of critical infrastructure in informal settlements across of Kampala city (Source: Adapted from geospatial datasets from Kampala Capital City Authority (KCCA), Makerere University Centre for Climate Change Research and Innovations (MUCCRI) and the Urban Action Lab (UAL), Makerere University).

**Figure 4:**
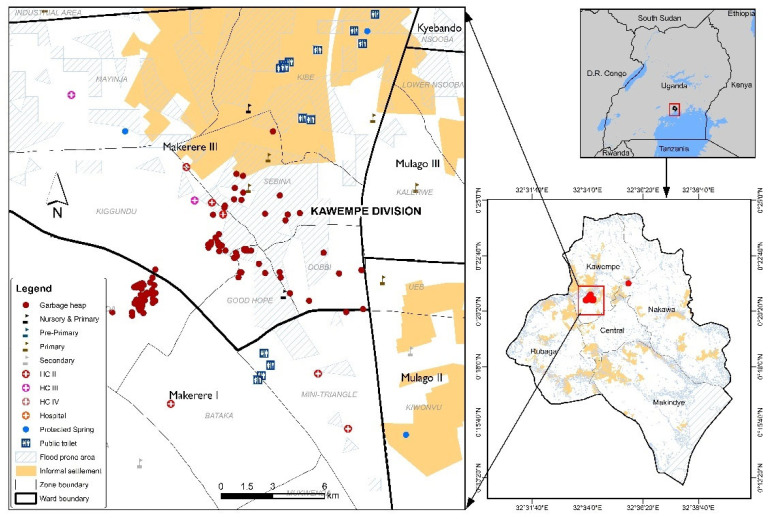
Geospatial Distribution of Illegal Garbage Dump Sites Alongside Critical Infrastructure (Schools, Health Facilities, Water, Sanitation and Hygiene points) and flood zones within Selected Settlements Zones in Kawempe Division.

The formative work consistently identified material deprivation as a defining feature of urbanicity in Kampala’s slums. Women emphasized poor sanitation (Figure 2), insecure housing, frequent flooding, accumulation of solid waste, and limited access to clean water and health services as central stressors. These environmental challenges created conditions of constant insecurity and uncertainty that shaped daily life and mental wellbeing as well as physical health [28]. Equally important was fragmentation, or the uneven juxtaposition of protective and harmful neighborhood features. Schools, water standpipes or churches might exist adjacent to bars, lodges, or poorly lit alleys (Figure 3 and Figure 4), producing environments where women have to negotiate risk and safety in close proximity.

There are clear manifestations of spatial fragmentation and deprivation in informal settlements of Kampala, coupled with climate change induced flooding vulnerability (Figure 3). Informality embodies stark realities of urban fragmentation and deprivation, particularly when viewed through the lens of critical infrastructure. The spatial organisation of informal settlements reveals significant disparities in access to essential services, which can exacerbate vulnerabilities and hinder community resilience. In Kampala, a highly fragmented urban landscape is prevalent, where critical infrastructure such as protected springs, public toilets, educational facilities, and health services are unevenly distributed. Informal settlements lie at the margins of urban planning, resulting in limited access to basic amenities. For instance, the presence of protected springs is sparse in these areas, and they continue to exist despite being banned by the KCCA. This scenario indicates a lack of safe drinking water sources, forcing residents, especially women, to rely on unsafe alternatives, which increase health risks and perpetuate cycles of poverty. Similarly, approximately 94% of residents in Kampala rely on on-site sanitation facilities that are not emptiable, while less than 10% of the city is connected to the piped sewer network. Currently, KCCA prohibited landlords from constructing on-site pit latrines throughout the city. This ban has created a significant service gap and heightened risks of poor air quality, particularly during dry seasons. The abandonment of full pit latrines and the discharge of faecal matter into drainage channels during rainy seasons exacerbate these risks, contributing to the potential spread of waterborne diseases such as cholera and typhoid. Public health facilities and educational institutions are also inadequately represented within informal settlements, The sparse distribution of schools highlight barriers to education, which present long-term implications for community development and social mobility. As an example, a public school in Bwaise III was submerged and has not been in use for over the past decade, raising psycho-social concerns for mothers and children due to risks associated with distant public education institutions. Similarly, public health facilities are often distant, leading to delays in medical care and worsening health inequalities. The geographical fragmentation of services reinforces the marginalization of informal settlement residents, hindering their ability to thrive.

Kampala, like many rapidly urbanising cities, faces a significant solid waste management crisis, a situation that worsened following the waste slide at the Kiteezi landfill, which led to its closure. The inadequacy of solid waste management has led to the proliferation of illegal dumpsites. Figure 4 revealed a troubling landscape of illegal dumpsites concentrated in various neighborhoods in the Kawempe division, particularly within informal settlements. These sites reflect limitations in municipal waste management systems and also the socio-economic realities of residents who often lack access to basic waste collection services. Poor waste management has led to the accumulation of waste in public spaces, drainage channels, and in toilet facilities, resulting in public health risks. Illegal dumpsites are often located in close proximity to residential areas and close to housing units, creating a hazardous environment. There are also currently no effective interventions for managing menstrual hygiene waste and child sanitary products, such as diapers. As a result, women, mothers, and young girls are often left with no choice but to dispose of these items in toilets or mix them with general solid waste at collection points. Women face exposure to unsanitary conditions, leading to health issues such as respiratory problems, water contamination, and vector-borne diseases, and yet the lack of effective waste management makes it difficult for communities to maintain a clean and safe living environment.

Drawing from Figure 3 and Figure 4, deprivation experienced in the informal settlements of Kampala city is multifaceted, encompassing economic, social, and environmental dimensions. Many residents, especially women, young girls, and children, face economic instability, making it challenging to invest in private alternatives for water, sanitation, solid waste management, and health services. The absence of reliable infrastructure and/or services exacerbates their vulnerability, particularly during extreme weather events, as inadequate and clogged drainage systems lead to flooding and habitat disruption. Moreover, the reliance on insufficient public facilities, such as communal toilets, often leads to public health risks. These facilities are frequently overburdened, poorly maintained, and some are abandoned due to their poor state, posing significant hygiene risks. The lack of integration between informal settlements and formal city infrastructure perpetuates a cycle of neglect, especially to the differential needs of women, leaving residents with no options but to navigate systemic barriers without adequate support. Together, these conditions illustrate how material deficits and highly variable access to services constitute a foundational dimension of how urbanicity is lived and experienced in Kampala’s slums.

### 3.2. Insecurity and Exclusion

**Figure 5:**
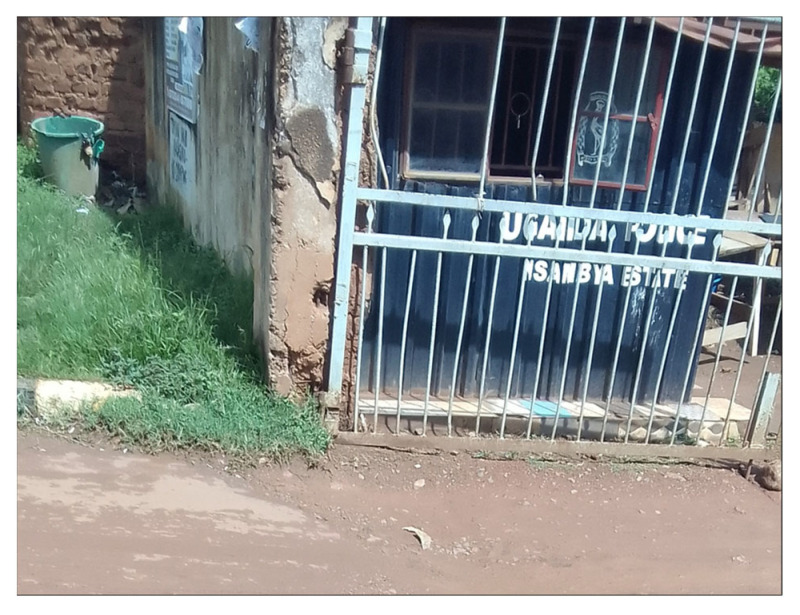
Picture of Nsambya Estate police post in Makindye division.

Safety concerns emerged strongly across both community mapping and Photovoice discussions. Women described experiences of harassment at boda boda stages, insecurity and safety concerns linked to alcohol outlets and gambling centers, and inadequate protection from overstretched or absent police posts. This sense of exclusion and vulnerability was not only physical but also institutional. Residents noted weak government presence, limited if not neglected service provision (Figure 5), and stigmatization of slum communities. Exclusion also reportedly amplified vulnerability to violence, trauma, and chronic stress, reinforcing feelings of marginalization and invisibility. These experiences demonstrate that exclusion and insecurity are not incidental but structurally embedded, shaping how women navigate their neighborhoods on a daily basis.

### 3.3. Protective and Resilience Factors

**Figure 6:**
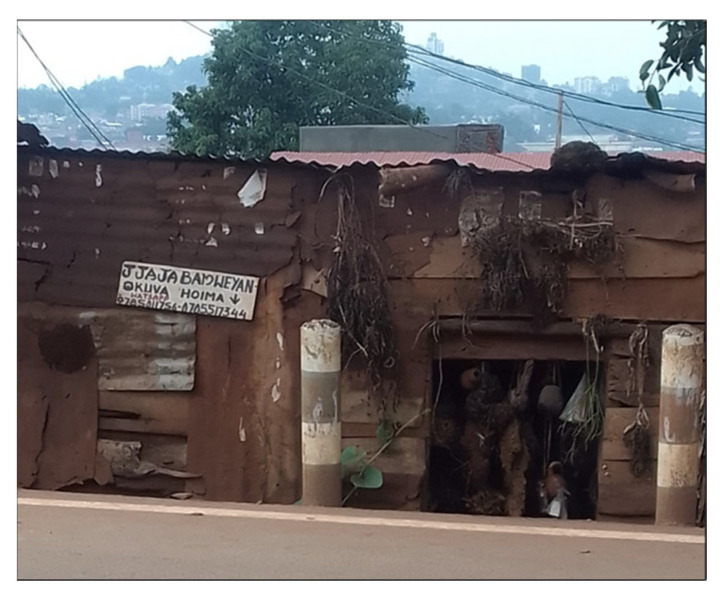
Picture of the Place of Workship in Banda—Nakawa Division.

Despite these challenges, protective features were consistently highlighted. Schools were valued as sources of hope, empowerment, and socialization, offering opportunities to pursue education, spaces for informal and non-structured knowledge/skills exchange, and catalysts for developing aspirations for the future. Places of worship (Figure 6) provided both spiritual guidance and psychosocial support, while health centers, though limited in capacity, offered a measure of security and access to essential care. Social networks, markets, youth groups, and sports activities were described as anchors of resilience, reinforcing collective survival strategies, neighborhood-level social and health welfare campaigns, and fostering a sense of belonging. Photovoice participants also emphasized adaptive practices such as small-scale farming and the use of green spaces, which provided both psychological restoration and nutritional resources. These protective environments highlight that resilience is woven into community institutions and social networks, even in contexts of significant deprivation and fragmentation.

### 3.4. Mechanistic Pathways Linking Urbanicity and Mental Health

Understanding how urbanicity shapes mental health requires moving beyond static descriptions of neighborhood features to tracing the processes through which these environments influence psychological outcomes. This requires analyzing multiple, interacting pathways through which neighborhood conditions shape psychological well-being. In Kampala’s slums, these pathways are neither linear nor simple, but are complex, cumulative, and reinforcing, producing layered vulnerabilities for women. Specifically, the convergence of environmental, social, political, and economic stressors creates layered pathways to depression, anxiety, and psychological distress. Evidence from community mapping and Photovoice demonstrates that these pathways are complex and often cumulative, involving interactions between structural disadvantage, locational-specific hardships, and individual vulnerability.

**Figure 7:**
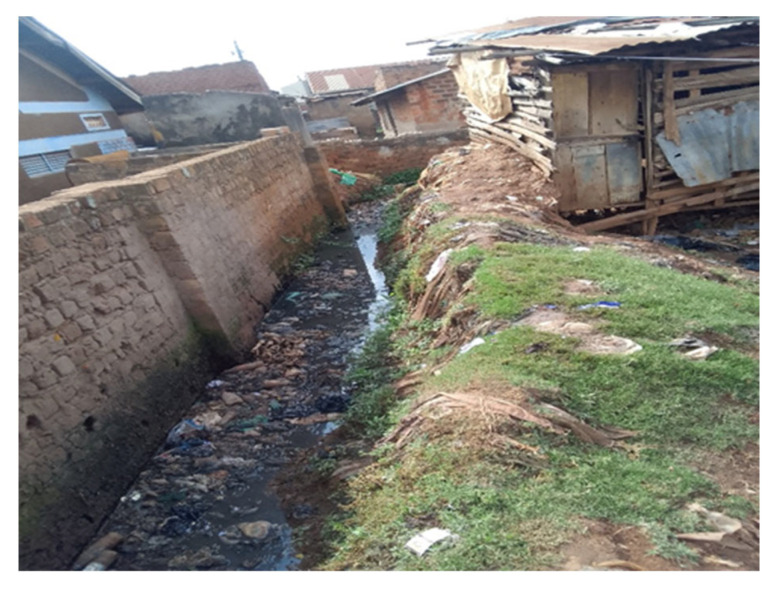
Photovoice submission of the urban living environment by: Esther, 21 years, Makindye.

One major pathway is chronic exposure to social environmental stressors, including the physical and social environment related to insecure housing and inadequate sanitation. Poor waste disposal, limited drainage, household congestion, and unreliable water supplies were consistently identified as defining features of daily life (Figure 7). These conditions create a backdrop of uncertainty in which families face heightened risk of illness, environmental hazards, and displacement. For young women, who often carry responsibility for caregiving and household management, the daily burden of navigating inadequate infrastructure compounds psychological stress and erodes resilience over time. Additionally, as demonstrated in earlier research, resilience is key to linking neighborhood context to mental health outcomes [30].

A second pathway involves exposure to violence and insecurity. Community mapping and Photovoice participants emphasized crime, poor security, gender-based violence, and alcohol-related harms as central neighborhood features. Bars, lodges, and alcohol outlets were described not only as prominent physical structures but also as symbols of pervasive risk environments. Alcohol availability intersected with poverty and social norms to create settings where violence against women, including intimate partner violence and sexual exploitation, became more likely. Trauma from these experiences was linked directly to distress, anxiety, and diminished well-being.

Substance use further intensified these risk pathways. Photovoice participants documented alcohol consumption and drug use as common activities associated with idleness and lack of employment opportunities. For many, alcohol use was viewed as both a coping strategy and a source of harm. Its use heightened vulnerability to violence, risky sexual behavior, and family conflict. Women described how these behaviors generated additional cycles of stress, leaving them to manage both the direct consequences of alcohol-related harms and the indirect effects on household functioning.

**Figure 8:**
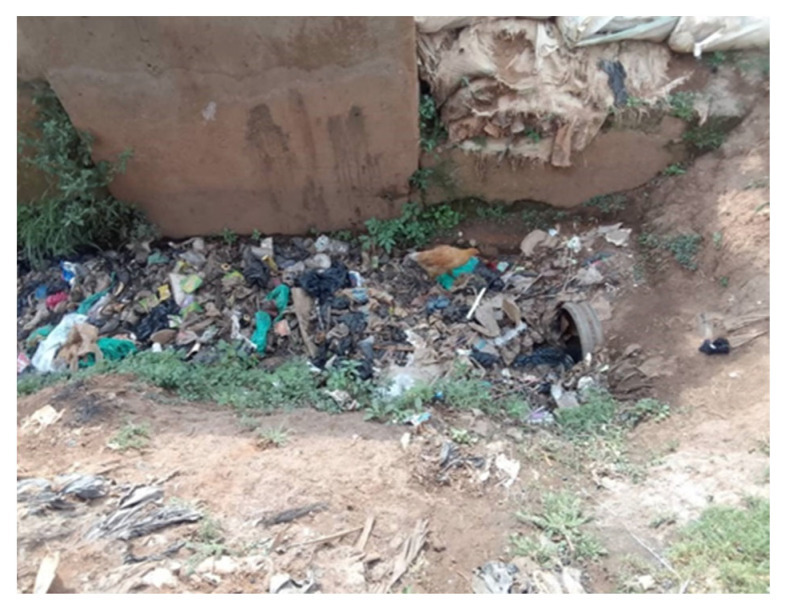
Photovoice submission of poor garbage handling and blocking the water drainage system by Shamie, age 24, in Bwaise.

Social environmental stressors were also amplified by climate-related challenges. Extreme heat, flooding, poor drainage (Figure 8), and unstable housing structures were described as routine features of life in slum settlements. Young women expressed significant climate-related anxieties, perceiving their neighborhoods as increasingly vulnerable to extreme weather events. These concerns were not abstract but grounded in lived experiences of property loss, health risks from contaminated water and uncollected waste, poor air quality, and the threat of displacement. The cumulative stress of managing both chronic poverty and environmental instability contributed to heightened anxiety and disrupted sleep, which itself is a critical pathway linking stress to poor mental health outcomes.

Social fragmentation constituted another important pathway. While communities displayed resourcefulness and creativity, mapping and Photovoice revealed the spatial separation of protective features, such as schools and places of worship, from risk environments, including bars, lodges, and poorly lit alleys. This fragmentation contributed to feelings of exclusion and weakened collective efficacy. For young women, exclusion from safe educational, employment, or recreational spaces deepened isolation and reduced opportunities to build supportive social networks, which are essential for resilience.

Biopsychosocial models and future research may help to integrate these observations by situating individual experiences within broader structural conditions. Physiological responses to chronic stress, including sleep disruption, changes in appetite, and dysregulation of mood, interact with social determinants such as poverty and gender-based violence to produce heightened vulnerability to depression and anxiety [15,16]. Cultural norms that devalue women’s voices and opportunities further constrain agency and reinforce cycles of risk.

Taken together, these mechanistic pathways highlight the need for a reconceptualization of urbanicity for young women in low-resource settings. Rather than treating urbanicity as a neutral demographic measure of density, it should be understood as a layered construct that actively produces risk environments with profound implications for women’s mental health. These interconnected mechanisms reinforce that urbanicity in informal settlements operates through layered and cumulative pathways rather than discrete risk or protective factors.

### 3.5. Conceptual Synthesis

From this formative evidence and building on our previous work, urbanicity in Kampala’s slums can be reconceptualized as a layered construct with neighborhood effects across four interrelated dimensions. Deprivation reflects material deficits in sanitation, housing, health, and services and infrastructure. Fragmentation refers to the spatial and social juxtaposition of protective and harmful environments within the same neighborhoods. Exclusion captures structural neglect, stigmatization, and limited institutional protection experienced by slum residents. Resilience encompasses the protective capacities embedded in education, faith, social networks, and adaptive community practices, and is key in linking neighborhood context and mental health outcomes [27].

This multidimensional framing reflects how urbanicity is lived and perceived by women themselves. It moves beyond conventional metrics of density or administrative categorization to capture the interplay of risks and resources that shape mental health in disadvantaged urban environments. These four dimensions, derived through participatory engagement, provide a grounded and context-specific framework for understanding urbanicity and neighborhood effects beyond conventional density- or infrastructure-based definitions.

## 4. Discussion

This formative work conducted in Kampala’s slums underscores the need to rethink urbanicity in ways that move beyond conventional definitions rooted in population density or the rural–urban divide. Evidence from community mapping and Photovoice reveals that women experience urban environments as multi-layered conditions defined simultaneously by deprivation, fragmentation, exclusion, and resilience. The conceptual model of neighborhood effects on mental health that emerges from this study suggests that urbanicity in slums can be reframed across four interrelated dimensions. These findings also highlight that urbanicity is not an abstract demographic variable, but a lived contextual experience embedded in daily struggles for safety, access to services and infrastructure, and opportunities for social participation, livelihood support, and economic prosperity As such, our findings build upon earlier work that challenged simplistic definitions of urbanicity, yet they diverge from these frameworks by grounding the concept in participatory, gendered, and spatial insights from informal settlements. The urbanicity experienced by young women in Kampala cannot be reduced to density, administrative status, or deprivation alone; instead, it is defined by the intersection of structural deficits, fragmented services, institutional absences, and community-driven forms of resilience.

Conventional frameworks for urbanicity have been shaped largely by research in high-income settings [31]. There, neighborhood studies emphasize indicators such as access to green space [35], noise pollution [32], or walkability as pathways to mental health [54]. Foundational theories, including social disorganization and collective efficacy, have advanced understanding of neighborhood effects, but they often fail to account for the structural realities of life in low-resource slums [40,55]. In Kampala, informal settlements are products not of gradual neighborhood decline but of rapid and unregulated urban growth combined with chronic poverty, limited governance, and structural neglect [29,48]. These differences call into question the validity of simply transplanting urbanicity measures from high-income contexts into low- and middle-income countries. Furthermore, recent work highlights that the mental health impacts of urban environments arise not only from density, but from the cumulative interaction of social, infrastructural, and environmental stressors across neighborhoods where poverty is a key driver [56,57]. In fact, there is growing empirical evidence from systematic reviews of global slum health that supports the need for conceptual frameworks of urbanicity that move beyond simplistic metrics of density or infrastructure, to capture the contexts of deprivation, fragmentation, exclusion, and resilience as core dimensions [58].

### 4.1. Deprivation: Material Constraints as Core to Urbanicity

Deprivation captures the material deficits of housing, sanitation, and essential services. Fragmentation reflects the spatial juxtaposition of protective and harmful features within the same communities, requiring residents to navigate risk and safety simultaneously. Exclusion emphasizes the structural marginalization of slum populations through weak government presence, stigmatization, and underinvestment in infrastructure. Finally, resilience highlights the protective features that residents themselves identify as critical, such as schools, religious institutions, health centers, sports activities, and community networks. Together, these dimensions provide a more comprehensive and contextually relevant understanding of urbanicity [59].

The mechanisms through which these dimensions influence mental health are cumulative and often mutually reinforcing. Chronic exposure to poverty, poor sanitation, flooding, violence, and harassment, among others, produces sustained stress that undermines psychological well-being [47]. Violence and alcohol-related harms exacerbate vulnerability to trauma [60,61,62,63], while social fragmentation erodes collective efficacy and increases isolation [64]. At the same time, protective features, especially education, worship spaces, and social networks, buffer against these risks and offer opportunities for resilience [29]. Conceptually, this layered model demonstrates that urbanicity in slum contexts cannot be reduced to static measures of density but should be understood as the interaction of structural risks and community strengths.

### 4.2. Fragmentation: Spatial Juxtaposition of Risk and Protection

The implications of this reconceptualization of neighborhood effects are significant for both research and practice. For researchers, it calls for methodological innovation. Reliance on census data (where available) or administrative categories obscures neighborhood heterogeneity in slums. Participatory approaches such as community mapping and Photovoice provide tools to capture local perspectives and ensure validity in how urbanicity is defined. Geospatial analysis, when combined with participatory input, can illuminate how risk and protective environments are distributed and experienced. Longitudinal designs are particularly valuable for tracing how changes in urban conditions affect mental health trajectories over time, while biomarker collection and sleep monitoring can link environmental exposures to physiological pathways, an approach we use in the TOPOWA study. In fact, recent findings from the TOPOWA cohort further demonstrates that mental health concerns among young women in Kampala’s slums remain highly prevalent and frequently co-occur with alcohol and drug use, highlighting the cumulative effects of deprivation, fragmentation, and exclusion in shaping wellbeing [65].

### 4.3. Exclusion: Institutional Absence, Neglect, and Powerlessness

For practice and policy, the framework highlights the importance of interventions that address both risk reduction and resilience building. Investments in sanitation, waste management, and secure housing and tenure are essential to reduce environmental stressors. There are evidence-based strategies for addressing aggressive alcohol marketing and availability, an issue of urgent concern given the frequency of exposure and the conceptualization of these marketing strategies as commercial determinants of health [66]. At the same time, interventions should leverage existing community strengths by supporting schools, health centers, youth organizations, and places of worship, all of which serve as anchors of resilience. Critically, these strategies should be developed with and through communities’ participation, ensuring that interventions align with lived experiences and community priorities.

Our findings resonate with feminist geographic arguments that urban environments are structured by gendered relations of power that shape how safety, mobility, and access to resources are distributed. Across Kampala’s slums, young women described negotiating public spaces in ways that reflect these gendered dynamics, including the need to manage harassment, avoid unsafe routes, limit movement at night, and accommodate caregiving expectations that restrict time and mobility. These gendered constraints illustrate that urbanicity cannot be understood solely through material or infrastructural indicators; it should be examined as a social and political condition that positions women differently within the same physical environment. Integrating these insights strengthens the argument that any reconceptualization of neighborhood effects in informal settlements should account for gendered experiences of risk, exclusion, and resilience.

Although this conceptualization is grounded in Kampala, several of the underlying processes we identify, rapid informal urbanization, inconsistent service provision, fragmented governance, and gendered vulnerability, characterize many cities across sub-Saharan Africa and parts of Asia and Latin America. We do not propose that the precise configuration of the four dimensions can be generalized without empirical grounding in other contexts. Instead, we offer this framework as a point of departure for comparative work that takes informality, gender, and lived experience seriously in understanding neighborhood effects on mental health. However, the association between urbanicity and depression observed in a recent global meta-analysis [67] reinforces the need for context-specific frameworks, as the mechanisms identified in formal urban environments differ in important ways from those operating within informal settlements. Yet research on urban health in low-resource settings remains limited, with earlier reviews noting that most studies focused on basic rural–urban comparisons or communicable diseases, leaving mental health and intra-urban inequalities in informal settlements largely underexamined [68]. Additionally, a recent regional review concluded that mental health research on adolescent girls and young women in Sub-Saharan Africa remains limited and uneven, despite clear evidence that a wide range of social, economic, neighborhood, and environmental determinants shape mental health across diverse settings [69].

### 4.4. Resilience: Social Networks, Aspirations, and Adaptive Practices

Finally, the Kampala case study illustrates the broader global significance of rethinking urbanicity. Rapid urban growth, climate change, and widening inequality are transforming cities across the Global South. Without new conceptual frameworks, public health research risks misrepresenting the realities of slum life and designing interventions that fail to resonate with local contexts. Reconceptualizing urbanicity across the dimensions of deprivation, fragmentation, exclusion, and resilience may provide a more accurate foundation for research and also serve as a practical guide for interventions. These findings also point toward opportunities for developing more contextually appropriate measures of urbanicity for informal settlements. Future work could draw on participatory spatial data to construct multidimensional indices that better capture environmental stressors, institutional presence, gendered risks, and community assets. Longitudinal designs, sensor-based environmental monitoring, and integration with biomarker data could further clarify pathways linking these dimensions to mental health. Such approaches would advance both the measurement and theoretical foundations of urban health research in low-resource settings.

This study has several limitations that reflect its primary purpose of developing a conceptual approach to rethink urbanicity and mental health in informal settlements. Rather than presenting a traditional empirical analysis, the manuscript synthesizes insights from three participatory sources—community mapping, Photovoice, and engagement with a community advisory board—together with findings from our prior research in Kampala. Because these data were generated through non-probabilistic sampling and conducted with young women engaged in UYDEL programs, the perspectives captured here do not represent all women living in Kampala’s informal settlements. The participatory activities documented neighborhood features that participants themselves viewed as most salient, meaning that not all spatial or environmental conditions were systematically mapped or considered. In addition, the issues emphasized in group discussions may have been shaped by dynamics inherent in participatory methods, including differences in social positioning, confidence, or familiarity with the tools. Despite these limitations, the triangulation of multiple participatory sources, combined with a decade of empirical research in these communities, provides a strong foundation for the conceptual framework proposed here. Future work using mixed-methods or quantitative approaches will be essential for testing and refining these conceptual dimensions across broader populations and settings.

## 5. Conclusions

Urbanicity has emerged as one of the defining determinants of health in the twenty-first century, yet its meaning and measurement remain contested. The experiences of young women living in Kampala’s urban slums reveal that urbanicity cannot be adequately understood as a demographic measure of population density or an administrative distinction between rural and urban. Instead, it should be defined as a multidimensional and dynamic construct shaped by deprivation, fragmentation, exclusion, and resilience.

In our case study, community mapping and Photovoice provided powerful insight into how residents themselves define the features of their neighborhoods that matter for wellbeing and mental health. Women consistently identified sanitation challenges, insecure housing, flooding, alcohol availability, violence, and poor institutional support as central risks. At the same time, they emphasized the importance of education, religious institutions, health services, and social networks as sources of resilience and hope. These insights demonstrate that urbanicity is experienced as both a set of structural risks and a foundation for adaptation and coping.

Rethinking urbanicity in this way has critical implications for global mental health research and policy. It can advance methodological rigor and innovation by grounding definitions in lived realities, strengthen causal inference, when applicable, by clarifying pathways between environments and mental health, and guide interventions that address both structural inequities and community resilience. For women in Kampala’s slums, interventions should move beyond physical infrastructure alone to include social protections, safe public spaces, participatory governance, and opportunities for empowerment.

This Kampala case study is emblematic of challenges faced in many rapidly urbanizing cities in the Global South, where informal settlements form faster than infrastructure, governance, or social protections can expand. Moving beyond narrow definitions of urbanicity is essential for the global health field to better capture the lived realities of slum life, design more effective interventions, and ensure that the voices of marginalized populations are central to knowledge production. Our findings demonstrate that urbanicity in informal settlements cannot be reduced to density, service availability, or deprivation alone. Instead, it should be understood as a layered and lived condition shaped by deprivation, fragmentation, exclusion and resilience, dimensions that simultaneously constrain and enable wellbeing. This framework holds promise for advancing methodological innovation, developing context-specific measures, and guiding interventions and policies that address structural risks while strengthening the community assets that make cities healthier, safer, and more equitable for their most disadvantaged residents. Ultimately, redefining urbanicity in this way is critical for improving mental health research, prevention, and care in the rapidly changing urban contexts that define the twenty-first century.

## Figures and Tables

**Table 1:** Community mapping indicators and Photovoice themes linked to mental health in Kampala’s urban slums.

Domain	Specific Features and Themes Identified
Sanitation and Environment	Poor disposal of solid wastes and poor sanitation; public latrines; drainage channels (poorly constructed and inadequate); toilets; wells and water sources; water drainage systems; nature; house congestion
Health and Services	Health centers and clinics; government hospitals; government schools; police posts; washing bays; sports facilities
Economic and Workspaces	Business and workplaces; jobs; markets; lodges; construction sites; farming; sports betting venues; video halls, streets
Social and Cultural Life	Worship places; bars and entertainment places; signposts; development infrastructure (electricity, transport networks); living lifestyles
Security and Risk	Alcohol selling points; idleness games and activities including drug and alcohol use and board games; security concerns, dark spots
People and Community	Children and women as focal subjects; community development features; youth participation; social support networks

## Data Availability

The data used from formative research is presented in this article. Additional information can be requested from Monica H. Swahn and be made available upon reasonable request.

## Abbreviations

The following abbreviations are used in this manuscript:

LMICs	low- and middle-income countries
TOPOWA	NIH-funded Onward Project on Wellbeing and Adversity
UYDEL	Uganda Youth Development Link
